# App-Based Mindfulness Meditation for People of Color Who Experience Race-Related Stress: Protocol for a Randomized Controlled Trial

**DOI:** 10.2196/35196

**Published:** 2022-04-14

**Authors:** Giovanni Ramos, Adrian Aguilera, Amanda Montoya, Anna Lau, Chu Yin Wen, Victor Cruz Torres, Denise Chavira

**Affiliations:** 1 Department of Psychology University of California, Los Angeles Los Angeles, CA United States; 2 School of Social Welfare University of California, Berkeley Berkeley, CA United States; 3 Department of Psychiatry and Behavioral Sciences University of California San Francisco San Francisco, CA United States; 4 Imperial Valley College Imperial Valley, CA United States

**Keywords:** race-related stress, discrimination, mindfulness, meditation, mental health, app, digital mental health intervention, racial and ethnic minority, people of color, BIPOC

## Abstract

**Background:**

People of color (POC) who experience race-related stress are at risk of developing mental health problems, including high levels of stress, anxiety, and depression. Mindfulness meditation may be especially well suited to help POC cope, given its emphasis on gaining awareness and acceptance of emotions associated with discriminatory treatment. However, mindfulness meditation rarely reaches POC, and digital approaches could reduce this treatment gap by addressing traditional barriers to care.

**Objective:**

This study will test the effectiveness of a self-directed app-based mindfulness meditation program among POC who experience elevated levels of race-related stress. Implementation outcomes such as treatment acceptability, adherence, and satisfaction will be examined.

**Methods:**

Participants (n=80) will be recruited online by posting recruitment materials on social media and sending emails to relevant groups. In-person recruitment will consist of posting flyers in communities with significant POC representation. Eligible participants will be block randomized to either the intervention group (n=40) that will complete a self-directed 4-week mindfulness meditation program or a wait-list control condition (n=40) that will receive access to the app after study completion. All participants will complete measures at baseline, midtreatment, and posttreatment. Primary outcomes include changes in stress, anxiety, and depression, and secondary outcomes constitute changes in mindfulness, self-compassion, rumination, emotion suppression, and experiential avoidance. Exploratory analyses will examine whether changes in the secondary outcomes mediate changes in primary outcomes. Finally, treatment acceptability, adherence, and satisfaction will be examined descriptively.

**Results:**

Recruitment began in October 2021. Data will be analyzed using multilevel modeling, a statistical methodology that accounts for the dependence among repeated observations. Considering attrition issues in self-directed digital interventions and their potential effects on statistical significance and treatment effect sizes, we will examine data using both intention-to-treat and per-protocol analyses.

**Conclusions:**

To our knowledge, this will be the first study to provide data on the effectiveness of a self-directed app-based mindfulness meditation program for POC recruited based on elevated race-related stress, a high-risk population. Similarly, meaningful clinical targets for POC affected by stressors related to race will be examined. Findings will provide important information regarding whether this type of intervention is an acceptable treatment among these marginalized groups.

**Trial Registration:**

ClinicalTrials.gov NCT05027113; https://clinicaltrials.gov/ct2/show/NCT05027113

**International Registered Report Identifier (IRRID):**

DERR1-10.2196/35196

## Introduction

### Background

In the United States, people of color (POC) are disproportionally affected by stressors related to race and ethnicity compared with their non-Latinx White counterparts, with approximately 70% of POC reporting having experienced some form of discrimination in their lives and 60% reporting facing day-to-day discriminatory treatment [1,2]. The prevalence of these discriminatory experiences varies across POC groups, with Black individuals (70%) experiencing more instances of discrimination compared with their Asian (57%) and Latinx (45%) counterparts [1]. Notably, approximately 60% of Black, 49% of Asian, and 45% of Latinx individuals state that these experiences make their lives harder [2]. Race-related stress is the psychological distress associated with appraising a situation as disturbing or burdensome because of negative racial bias [3]. Race-related stressors have multiple forms, such as direct interpersonal discrimination (ie, perceived differential and unfair treatment among individuals), vicarious discrimination (ie, observing members of one’s race or ethnicity being victimized), and institutional practices (ie, laws and policies that limit access to services and opportunities) [4,5]. Common race-related stressors experienced by POC include being treated with less courtesy or respect, receiving poorer service, receiving unfair treatment in the workplace, and negative interactions with police [2].

### Race-Related Stress and the Mental Health of POC

When differential or unfair treatment is perceived as racially motivated, it triggers physiological (eg, cardiovascular reactivity, dysregulated hypothalamic-pituitary-adrenal axis activity), cognitive-affective (eg, rumination, emotion suppression), and behavioral (eg, avoidance, aggression) responses that prepare the individual to confront the situation [6-8]. The chronic and unpredictable nature of race-related stressors often depletes individuals’ psychological resources and increases their vulnerability to developing mental health disorders [8]. Meta-analytic work shows that based on effect sizes, stress (*r*=.27), anxiety (*r*=.22-.25), and depression (*r*=.21-.26) are the mental health problems most strongly correlated with experiencing race-related stress, and these associations seem to be stronger among Asian and Latinx individuals compared with Black individuals [9-11]. This disproportionate exposure to race-related stressors may partly explain the higher prevalence and persistence rates of mood and anxiety disorders among Black, Asian, and Latinx groups compared with non-Latinx White groups [12,13]. These adverse mental health outcomes are especially concerning considering current social events, including instances of police brutality [14], recent political discourse [15], the COVID-19 pandemic [16], and an increase in hate crimes [17] that have made race-related stressors even more salient, further affecting the mental health of POC communities.

Considering POC exposed to race-related stress are at high risk of developing a mental health disorder, there is a clear need for treatments that allow individuals to cope effectively with these stressors. Among numerous evidence-based treatments available, mindfulness meditation may be particularly well suited to help POC cope.

### Mindfulness Meditation for Race-Related Stress in POC

Mindfulness is a psychological trait associated with staying in the present moment with one’s experience (ie, physical sensations, thoughts, emotions, behaviors), endorsing a nonjudgmental and curious attitude, and cultivating acceptance and self-compassion [18]. Self-compassion refers to an attitude of openness to one’s own suffering without avoiding it, cultivating the desire to alleviate it without self-judgment [19]. Multiple meta-analyses have shown that increases in mindfulness and self-compassion are two of the mechanisms by which mindfulness meditation leads to decreases in overall stress, anxiety, and depression [20-24].

Furthermore, mindfulness meditation could be particularly effective in helping POC cope with race-related stress by reducing experiential avoidance. Experiential avoidance is the inability to remain in contact with distressing physical sensations, thoughts, and emotions, even when doing so causes harm in the long term [25]. POC exposed to race-related stressors often develop emotional (eg, unwillingness to experience sadness after being discriminated against) and behavioral (eg, evading places or situations where discrimination is anticipated to occur) avoidance that maintain mental health symptoms [5,6,8]. As such, mindfulness meditation, with its emphasis on bringing awareness to all experiences regardless of their negative valance, may serve as an exposure strategy that reduces avoidance, leading to decreases in psychological distress [26].

Considering that rumination (ie, the passive and repetitive focus on the causes and consequences of one’s distress) [27] and emotion suppression (ie, the active reduction of emotionally expressive behavior when emotionally aroused) [28] are maladaptive emotion regulation strategies often used by POC exposed to race-related stressors [5,6,8,29], researchers and clinicians have hypothesized that mindfulness meditation may be particularly well-suited for this population [30-32]. Research suggests that mindfulness meditation disrupts ruminative tendencies by cultivating acceptance of and nonjudgmental engagement with thoughts [33]. In the case of emotion suppression, mindfulness meditation may help individuals identify, describe, and healthily engage with emotions triggered by race-related stressors rather than suppress these feelings, leading to decreases in mental health symptoms [34].

Although research to date supports the effectiveness of mindfulness meditation in reducing mental health problems, most studies have relied on almost exclusively non-Latinx White samples [35], which significantly differ from POC on critical demographic variables, such as level of education, income, and culture (ie, norms, language, beliefs, customs). For example, even in a meta-analysis where the primary aim was to examine mindfulness meditation’s effectiveness among underserved populations, less than 30% of studies included had POC samples [30]. A more recent systematic review surveying literature from 1990 to 2016 found only 24 studies examining the effectiveness of mindfulness meditation in POC samples, and just 25% of these studies were conducted with adults [36].

### Improving Access to Mindfulness Meditation for POC via Technology

Despite the significant mental health need among individuals who experience race-related stress and the existence of promising interventions such as mindfulness meditation, POC face numerous barriers to care, including limited access to providers [37], financial and transportation constraints [38], and stigma [39]. Although no single approach will eliminate all barriers driving mental health disparities, using technology to provide care could reduce this treatment gap [40,41].

Among many technological resources available, smartphones represent a promising vehicle for providing mental health services. The feasibility of this approach is supported by extensive ownership of mobile devices in the United States. For instance, approximately 85% of Americans have a smartphone, with POC having similar ownership rates as non-Latinx White individuals [42]. Compared with non-Latinx White people, POC are also more likely to be smartphone-dependent, meaning they rely on these devices to access the internet in the absence of a broadband connection at home. As such, interventions using smartphones may be well positioned to reach those affected by the digital divide observed in low-income households, as these interventions capitalize on technology already owned by POC [40,43].

In addition to their feasibility, there is a growing body of evidence supporting the efficacy of app-based mental health interventions [44,45]. Although somewhat limited, existing data with POC samples are promising [40]. In the case of app-based mindfulness meditation, multiple meta-analyses show that these interventions effectively reduce overall stress, anxiety, and depression [46-48] and increase mindfulness and self-compassion [47,48]. To our knowledge, experiential avoidance, rumination, and emotion suppression have not been examined as potential clinical targets in app-based mindfulness meditation. Furthermore, similar to face-to-face mindfulness meditation research, there is a lack of POC representation in app-based mindfulness meditation studies.

### Issues of Engagement in App-Based Mental Health Interventions

Despite their feasibility and empirical support, there are significant concerns regarding treatment engagement in app-based mental health interventions. Enrollment rates are low, with a meta-analysis showing that 8% to 58% of participants never download the intervention app [49]. Even when the app is downloaded, studies show that some users never use it, and those who engage with the app are unlikely to do so more than a few times, showing a decrease in use over time [49,50]. Similarly, attrition rates range from 24% to 53%, with higher rates found in longer programs (ie, more than 8 weeks) [49]. Importantly, programs without guidance (eg, via human coach, automated reminders) seem to lead to even poorer treatment engagement [51]. This limited treatment engagement in app-based mental health interventions is concerning as users may not receive enough treatment dosage to promote behavioral change.

Considering the importance of promoting treatment engagement for the success of app-based interventions, researchers and clinicians have proposed multiple strategies to increase acceptability, improve adherence, and reduce attrition. Among numerous approaches to promote app user engagement, cultivating a sense of guidance and support may be especially effective. Onboarding procedures in which users interact with providers before starting the intervention, asking questions about the intervention, problem-solving potential barriers to engagement, and building a relationship with the team have shown to be associated with higher treatment engagement [52]. Similar procedures in app-based interventions for POC seem to be effective as users can ask questions about the program and request assistance with technology [53]. As a whole, studies show that onboarding procedures decrease treatment attrition in app-based mental health interventions [49].

Among POC who have more limited access to technology, the use of text messaging notifications could be a especially well-suited strategy to increase treatment engagement. Text messages capitalize on the high phone ownership rates among POC while minimizing costs, as most phone plans include unlimited text messages. The feasibility of this approach is supported by previous studies showing that receiving simple text messaging reminders increases attendance to face-to-face treatment sessions among POC [54,55]. Importantly, this engagement strategy is well tolerated by participants [56]. These findings among POC are consistent with other studies showing that receiving prompts (eg, text messages, app notifications) leads to higher treatment engagement in digital interventions [57]. In app-based interventions, the use of prompts has also been found to be associated with lower attrition rates [49]. Therefore, exploring treatment acceptability, adherence, and satisfaction in these programs is crucial to determining whether mindfulness meditation programs are relevant for POC who experience race-related stress.

### This Study

Considering the lack of research examining the effectiveness of mindfulness meditation among POC, especially those who experience elevated levels of race-related stress, this study will use a randomized controlled trial (RCT) approach to examine whether receiving an app-based mindfulness meditation intervention leads to decreases in the adverse mental health outcomes more often associated with exposure to race-related stress (ie, stress, anxiety, depression) among POC. Similarly, this RCT will test whether the intervention engages hypothesized mechanisms of change (ie, mindfulness, self-compassion, experiential avoidance, rumination, emotion suppression). As an exploratory aim, this study will examine whether decreases in the primary outcomes of interest (ie, stress, anxiety, depression) occur via hypothesized mechanisms of change (ie, mindfulness, self-compassion, experiential avoidance, rumination, emotion suppression). Finally, acceptability, adherence, and satisfaction will be analyzed descriptively. Results from this trial will contribute to the nascent data on mindfulness meditation’s acceptability and effectiveness with POC. To our knowledge, this study will be the first to include a sample of POC recruited based on elevated levels of race-related stress, a high-risk population that is not commonly targeted in mindfulness meditation research.

## Methods

### Participants

A power analysis using G*Power [58] indicated that, based on 2 groups (ie, control vs active treatment), 3 repeated measurements (ie, baseline, midtreatment, posttreatment), a conservative *r*=.60 correlation among repeated measures, and a small *d*=0.2 treatment effect size, a sample of n=54 provides 95% power to detect a treatment by time interaction using the most conservative outcome in this study. Considering recent meta-analytic work suggesting an approximately 24% attrition rate in short-term (ie, 4 weeks or less) mindfulness meditation app trials with at-risk samples [49], we plan to recruit 80 POC individuals for this study.

Individuals interested in participating in this study will complete an online screening questionnaire using Qualtrics, a platform that is smartphone compatible. For study inclusion, participants are required to (1) self-identify as a member of a POC group (eg, Black/African American, Asian/Asian American/Pacific Islander, Latinx, Native American/Native Alaskan, multiracial, other), (2) report experiencing elevated levels of race-related stress (ie, a score of 55 or higher on the Index of Race-Related Stress–Brief [IRRS-B] [4] or a score of 12 or higher on the Multicultural Discrimination Module [MDM] [59]), (3) speak and read English, (4) not receive psychological services currently (ie, individual or group therapy, any type of counseling), (5) not have practiced mindfulness meditation for more than 2 hours in the month prior to study commencement, (6) own a smartphone with access to the internet, (7) be willing to install the mindfulness meditation app and accept daily notifications and text reminders, and (8) be aged 18 years or older.

Those individuals who meet eligibility criteria will be contacted by a research team member to obtain verbal consent and randomize them to either the intervention group (n=40) or a wait-list control group (n=40) using block randomization. During this onboarding procedure, participants in the intervention group will be assisted in installing the mindfulness meditation app, setting app notifications, understanding daily text messaging reminders, and getting familiar with the intervention content. Similar onboarding procedures have been shown to improve treatment adherence in app-based mindfulness meditation studies [49,51]. Participants in the control condition will be reminded of the scheduled assessments and when they will receive access to the mindfulness meditation app (ie, 4 weeks after randomization). Individuals who endorse self-harm or suicidal ideation during the onboarding procedure or in their replies to the text messages will be further assessed and referred to crisis support services.

### Ethical Approval

All procedures have been approved by the University of California, Los Angeles’ institutional review board (21-001426). This study was registered at ClinicalTrials.gov [NCT05027113].

### Procedures

Participants are being recruited through social media, sending emails to relevant groups, and posting physical flyers in communities with significant POC representation. Considering the current COVID-19 pandemic, data are being collected using an online procedure (ie, Qualtrics) at baseline (ie, randomization or week 0), midtreatment (ie, 2 weeks after randomization), and posttreatment (ie, 4 weeks after randomization) via online questionnaires. These assessments were piloted among POC to improve survey clarity and minimize participant fatigue before starting recruitment. These remote assessment strategies have shown to be effective in app-based intervention research with low-income POC [53].

**Figure 1:**
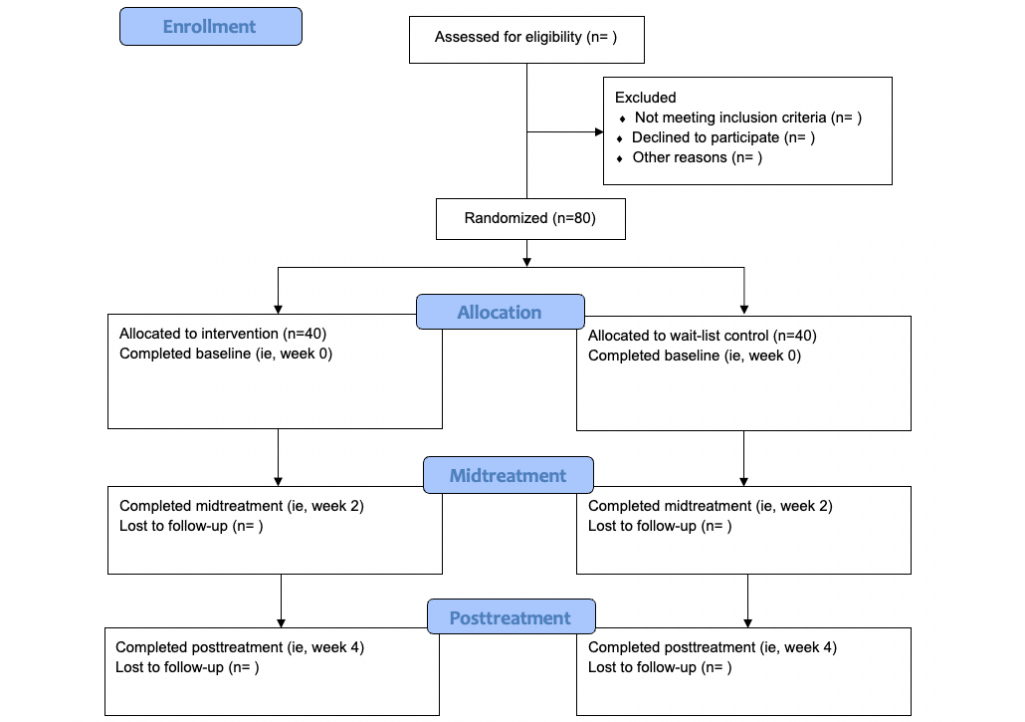
Study flow diagram.

Participants in both conditions will receive a free subscription to the mindfulness meditation app intervention as an incentive to participate. Additionally, participants will be compensated with US $10, $15, and $25 for completing baseline, midtreatment, and posttreatment questionnaires, respectively. There is evidence that monetary compensation significantly reduces attrition in app-based mindfulness meditation studies [49]. Figure 1 shows the study design flow.

### Mindfulness Meditation Intervention

This study will use the Ten Percent Happier app, a consumer-based mindfulness meditation app that offers a wide range of meditation practices developed and guided by renowned experts in the field. Among the many mindfulness apps available in the market, we chose this app because it has been shown to be an effective intervention to increase mindfulness and decrease anxiety and depressive symptoms in previous studies [60-62]. It is important to note that samples in these studies were almost exclusively non-Latinx White.

The intervention will consist of using the Ten Percent Happier app to complete at least 1 meditation daily—with the possibility of exceeding this goal by choosing additional meditations—for 4 weeks. More specifically, participants will be asked to complete The Basics and The Basics II courses to familiarize themselves with the principles of mindfulness meditation. Each audiorecorded session begins with a short video providing psychoeducation around mindfulness meditation and teaching specific techniques (eg, using the breath as an anchor, redirecting attention, noting thoughts and emotions). These 2 introductory courses comprise 15 sessions, in which the meditation length gradually increases from 5 to 17 minutes. For the last 2 weeks of the program, participants will be invited to complete the Essential Advice course, which dispels common myths around mindfulness meditation (eg, “meditators empty their minds,” “successful meditations are those without distractions”) and teaches participants how to deal with common obstacles in their practice (eg, difficult emotions, strong physical sensations). This additional course comprises 14 sessions ranging in length from 15 to 18 minutes. For any additional practice, participants will be able to select any meditation from the Ten Percent Happier library based on their preferences. This highly structured 4-week introductory course may be more likely to provide the foundation necessary for participants to benefit from mindfulness meditation compared with less structured programs often seen in app-based mindfulness meditation research [46]. Moreover, short-term app-based mindfulness meditation interventions (ie, 4 weeks or less) lead to lower attrition rates compared with longer programs [49].

Given the evidence of low treatment adherence in self-directed app-based mindfulness meditation [49-51], we will use a combination of app and text messaging reminders. The Ten Percent Happier app allows users to set a daily notification that can be personalized. Similarly, users will receive daily text messages reminding them to complete their daily meditation. Similar procedures have been hypothesized to foster a therapeutic relationship that keeps the user engaged and promotes a sense of accountability [50,52]. Text messaging reminders have proven effective in increasing treatment adherence among POC community samples [54-56,63], including those in app-based mindfulness meditation studies [49].

### Measures

#### Screening

##### Race-Related Stress

The 22-item IRRS-B [4] measures multiple sources of race-related stress, including individual, institutional, and cultural sources. Participants rate items on a scale ranging from 0 (this never happened to me) to 4 (this event happened and I was extremely upset), with higher scores indicating more race-related stress. Items include “You notice when POC are killed by the police, the media informs the public of the victims’ criminal record or negative information in their background, suggesting they got what they deserve,” “You have been subject to racist jokes by non-POC people in positions of authority and did not protest for fear they might have held it against you,” and “You were refused an apartment or other housing; you suspect it was because you are a POC.” The IRRS-B was developed with a POC sample (ie, Black individuals), showing adequate reliability (α=.78) for both cultural and individual subscales and acceptable reliability (α=.69) for the institutional subscale. The wording of the IRRS-B will be slightly adapted to capture the experience of multiple POC groups rather than only Black individuals. For study inclusion, participants need to obtain a score of 55 (range 0-88), which represents elevated race-related stress.

##### Perceived Discrimination

The 8-item MDM [59] measures perceived discrimination across different racial, ethnic, and cultural groups. Participants rate items on a scale ranging from 1 (never) to 4 (often), with higher scores indicating more levels of perceived discrimination. Items include “How often have you been treated with less respect than other people?” “How often have people acted as if they think you are not smart?” and “How often have you been threatened or harassed?” The MDM was developed using a sample representative of the US population (approximately 40% POC) and has shown good internal consistency (α=.81-.88) in a previous study [59]. For study inclusion, participants need to obtain a score of 12, which represents the 75th percentile of the population distribution.

#### Primary Outcomes

##### Stress

The 10-item Perceived Stress Scale (PSS) [64] is the most widely used measure of perceived stress. Participants rate items on a scale ranging from 0 (never) to 4 (very often), with higher scores indicating more stress. The measure includes items such as “How often have you felt that you were unable to control the important things in your life?” “How often have you felt confident about your ability to handle your personal problems?” and “How often have you been able to control irritations in your life?” The PSS has shown good reliability (α=.84-.86) and validity [64], and it is commonly used in mindfulness meditation research [22]. The PSS will be administered at baseline, midtreatment, and posttreatment.

##### Anxiety

The General Anxiety Disorder (GAD-7) scale [65] is one of the most widely used measures of anxiety symptoms. Participants rate items on a scale ranging from 0 (not at all) to 3 (nearly every day), with higher scores indicating more severe anxiety symptoms. The scale includes items such as “Feeling nervous, anxious or on edge,” “Not being able to stop or control worrying,” and “Feeling afraid as if something awful might happen.” The GAD-7 has shown good internal consistency (α=.89-.90) and validity in POC samples [66]. The GAD-7 will be administered at baseline, midtreatment, and posttreatment.

##### Depression

The 8-item Patient Health Questionnaire (PHQ-8) [67] is one of the most widely used measures of depressive symptoms. Participants rate items on a scale ranging from 0 (not at all) to 3 (nearly every day), with higher scores indicating more severe depressive symptoms. This measure includes items such as “Little interest or pleasure in doing things,” “Feeling down, depressed, or hopeless,” and “Feeling bad about yourself or that you are a failure or have let yourself or your family down.” Although the PHQ-8 omits an item assessing suicidal ideation and self-harm, meta-analytic work shows this version is practically equivalent to the original PHQ-9 measure [68]. The PHQ-8 has good reliability (α=.86-.89) and has shown validity across different racial and ethnic groups [69]. The PHQ-8 will be administered at baseline, midtreatment, and posttreatment.

#### Secondary Outcomes

##### Mindfulness

The 15-item Mindful Attention Awareness Scale (MAAS) [70] measures mindfulness. Participants rate items using a scale ranging from 1 (almost always) to 6 (almost never), with higher scores indicating more mindfulness. Items include “I find it difficult to stay focused on what’s happening in the present,” “I tend not to notice feelings of physical tension or discomfort until they really grab my attention,” and “I could be experiencing some emotion and not be conscious of it until sometime later.” The MAAS is one of the most used measures of mindfulness [71], showing good internal consistency (α=.80-.87) during its development [70]. The MAAS will be administered at baseline, midtreatment, and posttreatment.

##### Self-compassion

The 12-item Self-Compassion Scale–Short Form (SCS-SF) [19] measures self-compassion. Participants rate items using a scale ranging from 1 (almost never) to 5 (almost always), with higher scores indicating more self-compassion. Items in this measure include “I try to be understanding and patient toward those aspects of my personality I don’t like,” “I try to see my failings as part of the human condition,” and “When I’m going through a very hard time, I give myself the caring and tenderness I need.” In the development study of the SCS-SF, this scale showed good internal consistency (α=.86) and validity in a sample with significant POC representation [19]. The SCS-SF will be administered at baseline, midtreatment, and posttreatment.

##### Experiential Avoidance

The 15-item Brief Experiential Avoidance Questionnaire [25] measures experiential avoidance. Participants rate their responses on a scale ranging from 1 (strongly disagree) to 6 (strongly agree), with higher scores indicating more experiential avoidance. Items include “I’m quick to leave any situation that makes me feel uneasy,” “I would give up a lot not to feel bad,” and “I work hard to keep out upsetting feelings.” The BAEQ has shown good internal consistency (α=.80-.86) and construct validity [25]. The BAEQ will be administered at baseline, midtreatment, and posttreatment.

##### Rumination

The 5-item brooding subscale of the short version of the Ruminative Response Scale (RRS-SF) [27] measures rumination. Participants rate their responses on a scale ranging from 1 (almost never) to 4 (almost always), with higher scores indicating more rumination. Items include “Why can’t I handle things better?” “Why do I have problems other people don’t have?” and “What am I doing to deserve this?” The RRS-SF brooding subscale has shown adequate internal consistency (α=.79) and validity in studies with POC samples that experience discrimination [72]. This subscale will be administered at baseline, midtreatment, and posttreatment.

##### Emotion Suppression

The 4-item expressive suppression subscale of the Emotion Regulation Questionnaire (ERQ) [28] measures emotion suppression. Participants rate their responses on a scale ranging from 1 (strongly disagree) to 7 (strongly agree), with higher scores indicating more emotion suppression. Items include “I keep my emotions to myself,” “When I am feeling positive emotions, I am careful not to express them,” and “I control my emotions by not expressing them.” The expressive suppression subscale of the ERQ has shown adequate reliability (α=.68-.76) and validity in samples with significant POC representation [28]. Furthermore, this subscale has been used in mindfulness research with POC [29]. The ERQ will be administered at baseline, midtreatment, and posttreatment.

#### Descriptive Outcomes

##### Acceptability and Appropriateness of the Intervention

The Attitudes Toward Psychological Online Interventions (APOI) [73] is a 16-item measure of experiences with digital interventions with 4 subscales (ie, skepticism and perception of risks, confidence in effectiveness, technologization threat, anonymity benefits). Participants rate items on a scale ranging from 1 (totally agree) to 5 (totally disagree), with higher scores indicating more perceived acceptability and appropriateness. The skepticism and perception of risks subscale includes items such as “By using the app, I do not expect long-term effectiveness” and “It is difficult to implement the suggestions of the app effectively in everyday life.” The confidence in effectiveness subscales contains items such as “I have the feeling that the app can help me” and “I believe that the concept of app interventions makes sense.” The technologization threat subscale includes items such as “I am more likely to stay motivated with a therapist rather than when using an app” and “I learn skills to better manage my everyday life from a therapist rather than from an app.” The anonymity benefits subscale has items such as “An app is more confidential and discreet than visiting a therapist” and “I would be more likely to tell my friends than I use an app than that I visit a therapist.” The APOI has shown good reliability (α=.83) and validity in a previous study [74]. The wording of the APOI will be slightly modified to refer to app-based interventions rather than online interventions. The APOI will be administered to participants in the intervention group at baseline, midtreatment, and posttreatment.

##### Treatment Satisfaction

The 7-item satisfaction with therapy subscale of the Satisfaction with Therapy and Therapist Scale–Revised [75] assesses treatment satisfaction with the app program. Participants rate items on a scale ranging from 1 (strongly disagree) to 5 (strongly agree), with higher scores showing more treatment satisfaction. Examples of items include “I am satisfied with the quality of the therapy I received,” “My needs were met by the program,” and “I would recommend this program to a friend.” The satisfaction with therapy subscale has shown excellent reliability (α=.92) and validity in a digital intervention study with a racially and ethnically diverse sample [76]. The wording of the satisfaction with therapy subscale will be adapted to suit the app-based format of the intervention better. The satisfaction with therapy subscale will be administered to participants in the intervention group at baseline, midtreatment, and posttreatment.

##### Treatment Adherence

Number of meditations completed, number of days with at least one meditation completed, and total time meditated in minutes will be used as behavioral measures of treatment adherence. This information is tracked by the Ten Percent Happier app and will be obtained by asking participants in the intervention group to share a screenshot of their app profile via text message. Previous studies have shown that this methodology allows researchers to obtain accurate behavioral indicators of treatment adherence [60,77]. These behavioral indicators will be collected at midtreatment and posttreatment.

## Results

Recruitment began in October 2021. This study will use multilevel modeling, a statistical methodology that allows time-varying predictors and handling dependence among repeated observations. We will examine changes in primary outcomes (ie, stress, anxiety, depression), hypothesized mediators (ie, mindfulness trait, self-compassion, experiential avoidance, rumination, emotion suppression), and treatment effects from baseline to midtreatment and posttreatment, and from midtreatment to posttreatment within each group. Test of mean differences will be calculated by fitting models with group condition (ie, intervention vs control) as the predictor, time as a within-group factor (ie, baseline, midtreatment, posttreatment), and a group-by-time interaction. To explore whether changes in primary outcomes are mediated by changes in the hypothesized mechanism of change, we will conduct mediation analyses. Relevant demographic variables (eg, income, age, gender) will be added to the models as covariates. These models will include random intercepts to capture potential differences in the starting points of each outcome and an autoregressive error structure to account for dependence among repeated measures. Given the relatively small sample size in this study, random slopes will be fitted when possible. Although our primary interest is in group-by-time interactions corresponding to differential treatment effects, should significant interactions or main effects be found, follow-up contrasts can be used to examine within-group changes over time and between-group differences at each time point.

Considering issues of attrition and their potential effects on statistical significance and treatment effect sizes in app-based mindfulness meditation research [49], we will examine data using both intention-to-treat and per-protocol analyses [78]. In intention-to-treat analyses, group comparisons include all patients originally allocated after randomization, regardless of participant adherence to the intervention or withdrawal. Missing data will be handled using the last-observation-carried-forward method, which is considered a conservative approach. In per-protocol analyses, group comparisons include only those participants who completed the treatment as planned originally. Measures of treatment acceptability, satisfaction, and adherence will be examined by calculating descriptive statistics.

## Discussion

### Principal Findings

Despite extensive evidence showing the negative mental health effects of race-related stress in POC [6-11,14-17] and the promise of mindfulness meditation to help these individuals cope [20-24,26,30-32,79], this evidence-based treatment continues to be understudied among this high-risk population in both traditional [30,35,36] and digital formats [46-48]. As such, this study seeks to address gaps in the literature by (1) recruiting a sample exclusively composed of POC who report elevated levels of race-related stress and (2) testing the acceptability and effectiveness of a self-guided app-based mindfulness meditation intervention.

The main goal of this study is to determine whether POC who receive this self-directed app-based mindfulness meditation intervention experience reductions in mental health symptoms often associated with experiencing race-related stress. Based on previous meta-analyses [46,47,79], we hypothesize that POC who receive the intervention will experience significant decreases in overall stress, anxiety, and depression compared with those in the control group.

An additional goal of this study is to examine whether this self-directed app-based mindfulness meditation program engages potentially relevant clinical targets for POC exposed to race-related stress. Based on previous meta-analyses [20-24], we hypothesize that POC who receive the intervention will experience significant increases in mindfulness and self-compassion compared with those in the control group. Although increases in mindfulness are a well-established mechanism of change in mindfulness meditation programs, the role of self-compassion is less understood, warranting further investigation [20,21,23,24]. Considering previous studies showing the crucial role of experiential avoidance [80,81], rumination [20,23,24,82,83], and emotion suppression [29,83,84] as mediators in the development of mental health problems among individuals exposed to stressors related to race, we hypothesize that POC who receive the intervention will experience significant decreases in these 3 variables compared with those in the control group.

Although mediation analyses are exploratory in nature, given the relatively small sample in this study, we hypothesize that changes in the secondary outcomes (ie, mindfulness, self-compassion, experiential avoidance, rumination, emotion suppression) will mediate changes in the primary outcomes (ie, stress, anxiety, depression). These analyses will provide novel information regarding potentially meaningful mechanisms of change in mindfulness meditation interventions among POC exposed to race-related stressors.

Finally, information regarding treatment acceptability, satisfaction, and perceived appropriateness may be especially important, given researcher and clinician concerns regarding potential cultural mismatches between mindfulness meditation and the needs of POC [32,85]. Similarly, data on treatment adherence in self-directed app-based mindfulness meditation are crucial to understanding whether this digital approach can indeed reduce unmet mental health need among POC while capitalizing on technology already available in households with more limited technological device ownership [40]. Issues of treatment adherence are significant considering extensive evidence of low enrollment, poor use, and high attrition rates in self-directed app-based interventions [49-51]. As such, descriptive information regarding number of individuals enrolled versus those eligible, number of meditations completed, total time meditated in minutes, survey completion rate, and number of dropouts is essential to determine the feasibility of this type of program and the potential impact of the engagement strategies used in this study (ie, short program length, onboarding procedure, text messaging reminder system, app notifications, financial incentives).

### Conclusion

To our knowledge, this will be the first study to include a sample of exclusively POC presenting with elevated levels of race-related stress, contributing to the nascent data on the acceptability and effectiveness of self-directed app-based mindfulness meditation among marginalized groups. This study may help reduce disparities in mindfulness meditation among POC by testing an innovative delivery method and identifying relevant clinical targets for this at-risk population. Findings from this study could also inform the design of future studies using methodologies that provide more nuanced information about contextual and cultural factors influencing the success of digital mindfulness meditation interventions among POC (eg, qualitative, mixed methods). Similar approaches have provided relevant data on factors facilitating the provision of traditional face-to-face mental health services for communities of color [86]. This line of research is necessary to guarantee that app-based interventions for POC attend to issues of diversity, equity, and inclusion, reducing inequities already present in brick-and-mortar mental health services [43].

## Abbreviations

APOI: Attitudes Toward Psychological Online Interventions
ERQ: Expressive Suppression of the Emotion Regulation Questionnaire
GAD-7: General Anxiety Disorder
IRRS-B: Race-Related Stress–Brief
MAAS: Mindful Attention Awareness Scale
MDM: Multicultural Discrimination Module
PHQ-8: Patient Health Questionnaire
POC: people of color
PSS: Perceived Stress Scale
RCT: randomized controlled trial
RRS-SF: Ruminative Response Scale, short form
SCS-SF: Self-Compassion Scale–Short Form
